# Treatment of avulsion fractures of the pelvis in adolescent athletes: A scoping literature review

**DOI:** 10.3389/fped.2022.947463

**Published:** 2022-09-23

**Authors:** Fabrizio Di Maria, Gianluca Testa, Fabio Sammartino, Marco Sorrentino, Vincenzo Petrantoni, Vito Pavone

**Affiliations:** Department of General Surgery and Medical Surgical Specialties, Section of Orthopaedics and Traumatology, University Hospital Policlinico “Rodolico-San Marco”, University of Catania, Catania, Italy

**Keywords:** adolescent athletes, sport lesion, avulsion fracture, pelvis, treatment, outcome

## Abstract

Avulsion fractures of the pelvis and hip region are typical injuries in adolescent athletes. Avulsion sites include the muscle tendon origin or insertion, and treating these injuries surgically or conservatively remains a debated issue. The main goals of this review were to assess and summarize injury types and sites, treatment-related clinical outcomes, and return to sport for adolescent patients with a pelvis avulsion fracture and to provide support for making treatment decisions. The PubMed database was searched in November 2021 to identify all published articles from 2000 to 2021 that reported the outcome and return to sport after conservative or surgical treatment. Eighteen studies with 453 patients were included in this review. The age range was 13.6–16.8 years. The most common injury site the was anterior superior iliac spine (37%), followed by the anterior inferior iliac spine (31%), ischial tuberosity (14%), lesser trochanter (9%), iliac crest (8%), and superior corner of the pubic symphysis (1%). Overall complications were lower in the surgical group compared to the conservative group. The rate of return to pre-injury activity level was greater in patients who underwent surgical treatment (*p* < 0.05). In conclusion, surgery is preferred for major dislocation and fragment size, providing a better return-to-sport rate and decreasing the risk of complications.

## Introduction

Adolescent sport injuries have increased in frequency and in importance over the last 2 decades, and 3–5% of these injuries involve the groin region (1). Among them, apophyseal avulsion fractures of the pelvis are caused by forceful muscle contraction or sudden and excessive passive muscle stretching, and they typically occur in young athletes whose cartilaginous growth plates are not ossified (2). Until the cartilage tissue is replaced, the secondary ossification centers are weaker than the muscular–tendinous unit, which is why avulsion fractures are more likely to occur than a tendon or muscle injury (3). Compared to other musculoskeletal trauma in adolescents, most patients (68.5%) with pelvic avulsion are male (4). This type of injury occurs primarily in sports that require quick change of direction, running, jumping, and other athletic movements that require intense and sudden strain including sports such as skiing, soccer, American football, boxing, track and field, and ice hockey (5).

The pelvic sites that are most frequently involved are the main bundle of the rectus femoris insertion at the anterior inferior iliac spine (AIIS), the sartorius insertion at the anterior superior iliac spine (ASIS), the hamstring insertion at the ischial tuberosity (IT), the tensor fascia latae on the iliac crest (IC), the rectus abdominis insertion on the superior corner of the pubic symphysis (SCPS), and the iliopsoas insertion on the lesser trochanter (LT).

Patients usually report feeling an abrupt “crack” and pain onset in the pelvic region that is more severe upon exercise and decreases with rest (6).

A standard radiograph a few days after the injury is usually sufficient to make a correct diagnosis. However, if there is any doubt about the diagnosis or if it has been several days since the onset of symptoms, other investigative modalities such as computed tomography and magnetic resonance imaging can confirm the diagnosis and assist with planning treatment (7). However, the use of a conservative or surgical approach remains controversial.

Non-surgical therapy with symptomatic treatment of pain and gradual resumption of activity are usually recommended (8). However, mini- or low-invasive surgical techniques such as K-wires, arthroscopy, percutaneous fenestration with plasma rich platelets, or open reduction and internal fixation with screws have been increasingly used (9–11), and the key element for the choice of treatment is the degree of fragment detachment (12).

This review aims to assess and summarize the type and site of the injury, treatment-related clinical outcomes, and return to sport in adolescent pelvis avulsion fracture patients and to provide support for making treatment decisions.

## Materials and methods

Our search was conducted in accordance with the Preferred Reporting Items for Systematic Review and Meta-Analyses (PRISMA) guidelines (13).

The electronic medical database PubMed was searched in November 2021 using the Boolean operators (AND, OR, and NOT), which were appropriately combined with the relevant terms in the following search string (“child” OR “adolescent”) AND (“pelvic avulsion” OR “avulsion fracture”) AND (“treatment” OR “therapy”) NOT (“dental”). Retrieved articles of any type, evidence level, and sample size were rated as eligible for review if the English full text was available. Studies from 2000 to 2021 were searched. Reviews, case reports, and articles on pelvic girdle fractures caused by a traumatic accident, avulsion fractures in other areas, and studies on non-sporting or adult populations were excluded.

Three reviewers (MS, FS, and FDM) independently screened all retrieved articles by title and abstract in accordance with the defined inclusion and exclusion criteria using a two-phase Delphi process. The full text of the articles that met the inclusion criteria was collectively reviewed by the reviewers. References within the included studies were also searched to identify articles that were missed by the initial search.

Data from the included articles (patient demographics, cause, and circumstance of injury, treatment decision, length of follow-up, clinical outcome, complication rate, and proportion of athletes returning to sport) were extracted in Excel format and verified by a fourth reviewer (VPe). Return to sport was defined as the rate of patients who had resumed their pre-injury level of sport at follow-up.

Statistical analysis was performed, using GraphPad (San Diego, CA, US). The Fisher's exact test was used to compare the return-to-sport rate between the surgical and conservative treatment groups. The selected threshold for statistical significance was *p* < 0.05.

## Results

**Figure 1:**
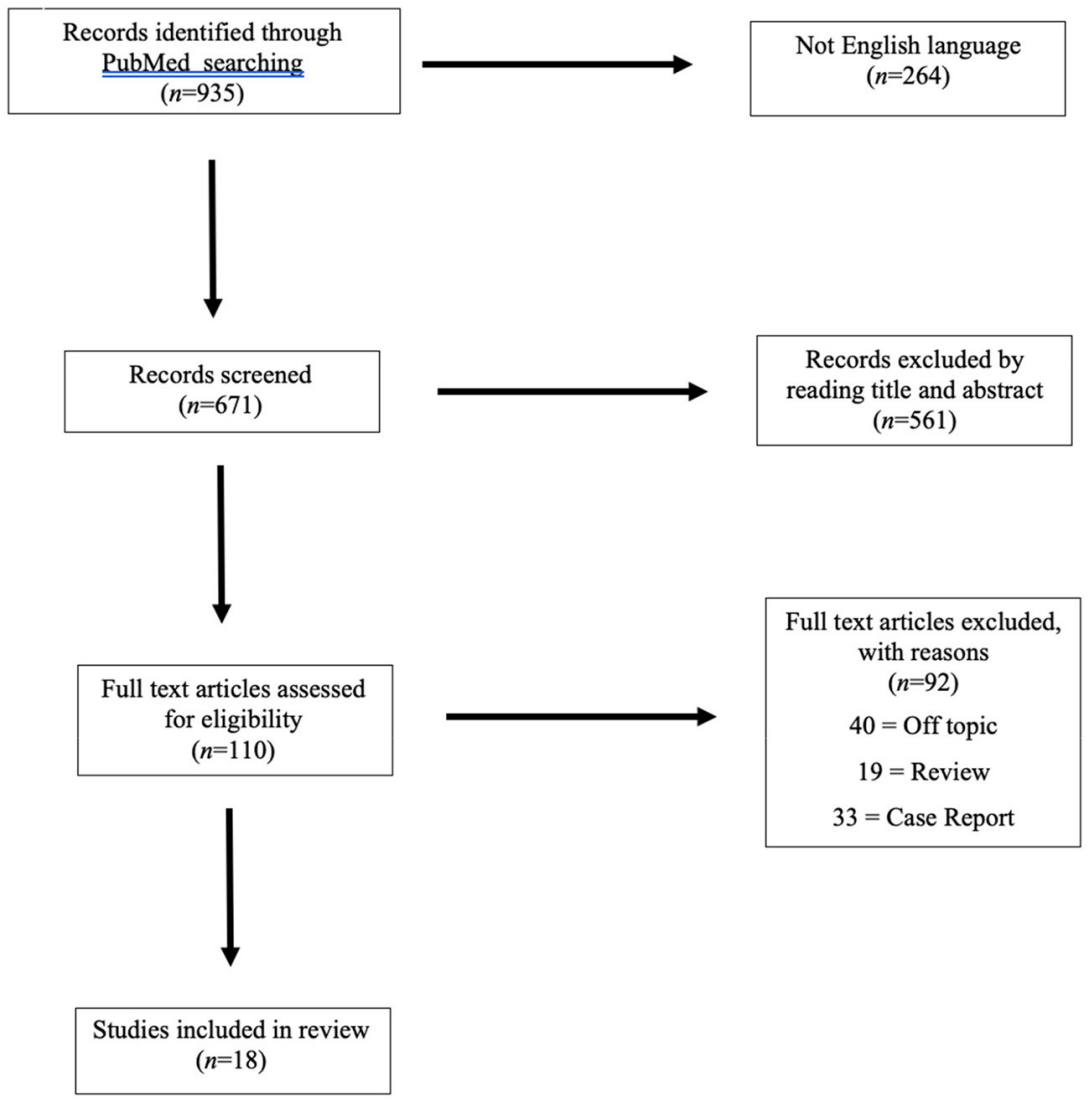
PRISMA (Preferred Reporting Items for Systematic Reviews and Meta-Analysis) flowchart of the systematic search.

**Figure 2:**
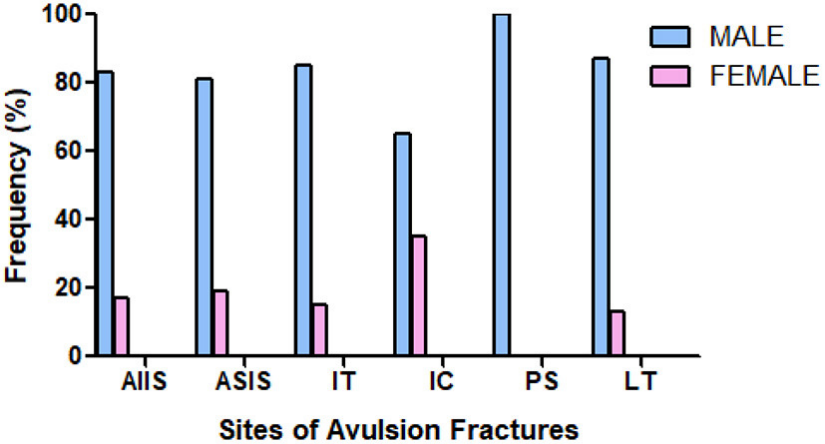
Histogram of prevalence by gender in site of avulsion fracture.

**Figure 3:**
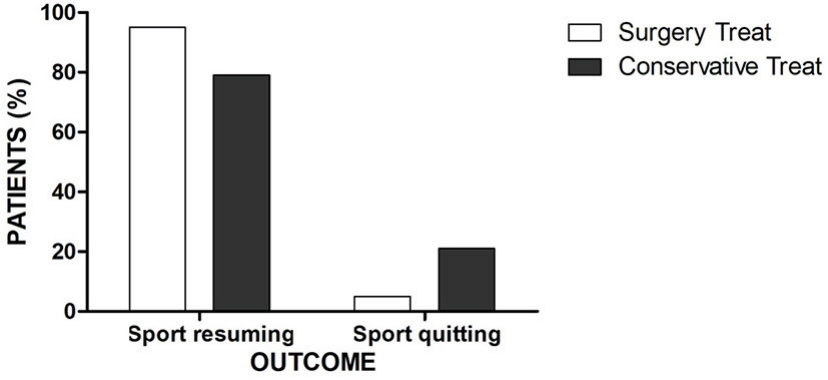
Histogram of rate to return to sport in surgical and conservative group.

Among the 935 articles identified through the initial search, 18 studies met the inclusion criteria (8–11, 14–27). A PRISMA flow-chart summarizing the selection pathway is shown in Figure 1. Four hundred fifty-three patients (82% males and 18% females; age range, 13.6–16.8 years) were included in this review. The patients' demographic information and study characteristics are shown in Table 1. The most common sites of injury were ASIS (37%) and AIIS (31%) followed by IT (14%), LT (9%), IC (8%), and SCPS (1%). Avulsion site prevalence by sex is shown in Figure 2. Among the included patients, 129 (28%) and 324 (72%) underwent surgical and conservative treatment, respectively. A return to the pre-injury level of sport occurred in 83% of the athletes overall. Return to sport occurred in 95% of patients who that underwent surgical treatment and 79% of patients in the conservative group, which was a significant difference (*p* = 0.0012; Figure 3).

**Table 1:** Patient's demographics and study characteristics.

**References**	**N. patients**	**Mean age**	**Gender, F/M**	**Site of injury**	**Treatment ST/CT**
Kosanovic et al. (17)	6	16	0/6	ASIS	6/0
Ferlic et al. (23)	13	14.7	1/12	IT	5/8
Li et al. (26)	10	14.6	1/9	IC	10/0
Uzun et al. (15)	9	14	0/9	AIIS	0/9
Khemka et al. (11)	3	15.3	0/3	LT	3/0
Schoensee et al. (10)	3	14.6	1/2	IT	0/3
Pogliacomi et al. (19)	9	16	2/7	6 ASIS 3 AIIS	9/0
Kautzner et al. (9)	23	15.1	4/19	ASIS	13/10
Biedert et al. (24)	3	14.4	0/3	IT	2/0
Serbest et al. (14)	5	13.6	2/3	AIIS	0/5
Schuett et al. (8)	228	14.4	55/173	112 AIIS 68 ASIS 25 IT 23 IC	0/228
Willinger et al. (18)	2	16.5	0/2	ASIS	2/0
Sinikumpu et al. (27)	32	16.8	2/30	11 AIIS 11 IT 5 SCPS 4 ASIS 1 IC	32/0
Ruffing et al. (20)	5	13.8	1/4	LT	0/5
Otto et al. (22)	2	14	0/2	LT	2/0
Cai et al. (16)	59	13.7	8/51	ASIS	30/29
Best et al. (25)	11	14.7	1/10	IT	11/0
Volpi et al. (21)	30	14.2	4/26	LT	0/30

A variety of complications, including meralgia paraesthetica, heterotypic ossifications, ossificans myositis, non-unions, and persistent hip pain, occurred in 118 (26%) patients overall. There was a lower frequency of complications in the surgical group (20%) compared to that in the conservative group (28%). Surgical failure was reported in only one patient.

## Discussion

This review showed that the most frequent pelvic avulsion fracture site in adolescent athletes was the ASIS and AIIS, which accounted for more than two-thirds of the avulsion fractures, while SCPS fracture was the least common (approximately one out of 100). Several factors could be responsible for these differences, the most important of which is related to the force of the muscles involved. Awareness of these injuries has increased over the past few years due to improved musculoskeletal imaging techniques and increasing participation of adolescents in competitive sporting activities. However, the non-specificity of reported symptoms results in a high risk of misdiagnosis, including muscles strain and bone bruising (28). Sex-related factors that are responsible for the greater frequency of sport-related avulsion fracture in adolescent males compared to females has not been fully investigated.

There are currently no guidelines for the best treatment of pelvic avulsion fractures. The lack of uniform and accepted criteria may contribute to the variability in the treatment decisions.

The treatment approach usually depends on the avulsion site and the amount that the avulsed segment is displaced. Therefore, conservative treatment, consisting of analgesics, limited activity, partial weight bearing for an average of 4–6 weeks, and a rehabilitation program, is considered the best treatment in non-displaced fractures (14, 15, 20, 21). However, potential complications may be associated such as chronic pain in the region of the avulsion fracture, non-unions, heterotopic ossifications, and a significantly decreased ability to return to pre-injury level activity. Ferlic et al. reported that pseudoarthrosis developed in half of the conservatively treated patients with an IT displacement of more than 15 mm (23).

For surgical treatment, no single cut-off was assessed for displacement of the avulsed segment, and multiple surgical techniques have been described for these fractures. Pogliacomi et al. used a dislocation of >20 mm as a cut-off, whereas Ferlic et al. and Cai et al. were more restrictive, with a 15 mm cut-off (16, 19, 23). Excellent outcomes were reported in most patients who underwent surgical treatment, such as a lower frequency of complications and a significantly higher return-to-sport rate compared to the conservative group.

Serbest et al. (14) analyzed a series of five cases with AIIS avulsion fractures that were all treated conservatively with bed rest. The affected hip was flexed at 90° for the first 2 weeks, total weight-bearing after 6 weeks, and complete return to sport at 10 weeks after injury. They reported a total recovery of the range of motion and a full return to pre-injury sports activity levels with excellent outcome and absence of complications. Similar outcomes were shown by Uzun et al. in nine male patients who were treated conservatively for AIIS avulsion (15).

Cai et al. (16) retrospectively analyzed 59 cases of patients with ASIS avulsion with diastasis >1.5 cm and compared the following two groups: the first group consisted of 29 patients who were treated conservatively (3 weeks of bed rest, hip 70°-90° of flexion followed by gradual loading); and the second group of 30 patients who were treated surgically using a resorbable screw (3–10 days bed rest, after loading with crutches). Return-to-sport activity occurred earlier in the surgical compared to the conservative group. Three patients in the conservatively treated group had myositis ossificans, which was not present in any of the surgical group patients. *Meralgia paresthetica* occurred in one and eight patients in the surgical and conservative groups, respectively. These results are comparable with surgical and conservative approaches that were performed in 23 patients with ASIS avulsion (9). Six weeks after treatment, a complete hip range of motion was achieved in ten out of 13 surgically treated patients compared to four of 10 conservatively treated patients (76.13 vs. 40%). Return to sport in the surgical and conservative group was 16 and 17 weeks, respectively. Two patients had to change their physical activity level because of pain and functional limitations after conservative treatment, whereas only one patient had to give up physical activity after surgical treatment. There were no major complications in these two groups. Optimal results with a complete return to pre-injury sports activity in the absence of complications was also observed in studies by Kosanovic et al. and Willinger et al. in patients with avulsion of the ASIS that was treated surgically using either Kirschner wires or a wire loop and that was anchored with non-resorbable sutures, respectively (17, 18).

Pogliacomi et al. (19) reported a cohort of nine adolescent athletes who were affected by ASIS or AIIS avulsion fractures with fragment displacement >2 cm and treated using screw osteosynthesis followed by immobilization for 30 days and a progressive return to sport 9 weeks after injury. All of these patients returned to their preinjury sport level, and only two developed transient meralgia paraesthetica.

Ruffing et al. (20) reported five adolescents with LT avulsion, all of whom underwent non-operative treatment and full-weight bearing at 6 weeks. All patients achieved the maximum Harris Hip Score (HHS) and each returned to practicing sports at a competitive level. Similar results were observed in Volpi et al.'s retrospective study of 30 patients who were treated conservatively (21). All patients returned to sport after an average of 11 weeks, and there were no complications reported.

Khemka et al. and Otto et al. independently described two innovative surgical techniques, arthroscopy fixation and open reduction, which both had an anterior approach. Patients had an excellent outcome and returned to sports after 10–12 weeks (11, 22).

Ferlic et al. (23) compared the results of surgical and conservative treatment in a retrospective series of 13 patients with IT avulsion, and the decision to perform surgery was based on radiologic evidence of fragment dislocation >15 mm. All surgically treated athletes had an excellent outcome with complete return to sport activity at 6 months. In the conservative group, the authors found a good outcome in all patients who had a fragment dislocation of <15 mm. In four patients with a dislocation fragment >15 mm, 50% reported the development of pseudoarthrosis and a significantly reduced ability to return to their sport.

Satisfactory results were obtained in Biedert's study of three patients with an IT avulsion that was treated surgically using three anchors (24). All patients resumed their pre-injury sports activities without restriction. Best et al. surgically treated 11 adolescent athletes, but long-term complications developed including failure to resume pre-injury sports activities in three patients (25).

Schoensee and Nilsson (10) analyzed a series of three cases in which the patients had chronic IT avulsion fractures. Percutaneous fenestration combined with conservative rehabilitation was used, which may have a significant therapeutic effect for adolescents whose early diagnosis is missed. None of the three patients subsequently showed complications or functional limitations.

Li et al. presented a series of ten patients who were treated surgically for IC avulsion fractures (26). These patients underwent fracture fixation using cannulated screws, which enabled immediate and active rehabilitations 2 days after surgery. These patients showed recovery of full athletic activities 4 weeks after the injury, and no complications were mentioned. Sinikumpu et al. retrospectively reviewed 32 adolescents who participated in sports and who had a variety of pelvic avulsion fractures (27). These patients underwent surgical treatment regardless of the distance of fragment displacement. Among them, 26 of 32 resumed their pre-injury sports. Post-surgical complications were reported in 22 patients, but among them, only six had to abandon competitive activity, and no significant difference was found between patients with more and <2 cm of displacement.

A large retrospective study including 228 cases was conducted by Schuett et al. (8). In this study, all injuries were managed conservatively with excellent outcomes in almost all patients. However, 14% of patients had recurrent or continued pain lasting more than 3 months especially in those with AIIS fractures. A high rate of non-unions (16%) was observed in IT avulsion with a displacement >2 cm (8).

All studies analyzed in this review reported excellent clinical outcomes in both the surgical and conservative groups. However, in most cases, the indication for conservative treatment was provided for a small displacement and fragment size, and a real comparison between these two groups was not possible.

All the authors agree that the physical demands on athletes who require rapid rehabilitation is another significant factor in the decision for or against surgery. This review showed that the surgically treated group had a significantly better return to sport at their pre-injury level compared to the conservatively treated group (Figure 3), especially for patients who had a fragment displacement >15 mm. An earlier return to sport due to the stable fixation and a decreased pain level may allow athletes to preserve their thigh muscle strength and cardiorespiratory endurance and thus, shorten the time to achieving their pre-injury performance.

Finally, this review showed that many surgical techniques exist to treat avulsion fractures of the pelvis, but there is no consensus on which treatment method has the best results. This could be a topic to investigate in the future.

Some limitations exist within the study. Only English language papers were included for this search, which may have excluded some studies of pelvis avulsion fractures. Additionally, the included studies had small sample sizes, which may be partially explained by the low incidence of these pathologies. Thus, prospective, randomized, controlled trials are required to answer the questions raised in this research.

## Conclusion

Avulsion fracture of the pelvis and hip region are typical injuries in male adolescent athletes. This review summarized the injury type and site as well as the clinical outcomes and return to sport in adolescents with a pelvis avulsion fracture who were treated surgically or conservatively. Both treatments provided excellent outcomes in most avulsion fractures. However, there was a higher return-to-sport rate and a lower frequency of complications in patients treated surgically compared to patients in the conservative group.

## Data availability statement

The original contributions presented in the study are included in the article/supplementary material, further inquiries can be directed to the corresponding author.

## Author contributions

FS, MS, and VPe treated the trauma aspects. FD and GT wrote the original manuscript. VPa revised the manuscript. All authors contributed to the article and approved the submitted version.

## Conflict of interest

The authors declare that the research was conducted in the absence of any commercial or financial relationships that could be construed as a potential conflict of interest.

## Publisher's note

All claims expressed in this article are solely those of the authors and do not necessarily represent those of their affiliated organizations, or those of the publisher, the editors and the reviewers. Any product that may be evaluated in this article, or claim that may be made by its manufacturer, is not guaranteed or endorsed by the publisher.

## References

[1] Morelli V Smith V. Groin injuries in athletes. Am Fam Physician. (2001) 64:1405–14. 11681783

[2] Porr J Lucaciu C Birkett S. Avulsion fractures of the pelvis - a qualitative systematic review of the literature. J Can Chiropr Assoc. (2011) 55:247–55. Available online at: http://www.ncbi.nlm.nih.gov/pubmed/22131561%0Ahttp://www.pubmedcentral.nih.gov/articlerender.fcgi?artid=PMC3222700 22131561 PMC3222700

[3] Sanders TG Zlatkin MB. Avulsion injuries of the pelvis. Semin Musculoskelet Radiol. (2008) 12:42–53. 10.1055/s-2008-1067936 18382943

[4] Soprano J V. Musculoskeletal injuries in the pediatric and adolescent athlete. Curr Sports Med Rep. (2005) 4:329–34. 10.1097/01.CSMR.0000306295.49707.1f 16282035

[5] Rossi F Dragoni S. Acute avulsion fractures of the pelvis in adolescent competitive athletes: prevalence, location and sports distribution of 203 cases collected. Skeletal Radiol. (2001) 30:127–31. 10.1007/s002560000319 11357449

[6] Moeller JL. Pelvic and hip apophyseal avulsion injuries in young athletes. Curr Sports Med Rep. (2003) 2:110–5. 10.1249/00149619-200304000-00011 12831668

[7] Singer G Eberl R Wegmann H Marterer R Kraus T Sorantin E. Diagnosis and treatment of apophyseal injuries of the pelvis in adolescents. Semin Musculoskelet Radiol. (2014) 18:498–504. 10.1055/s-0034-1389267 25350828

[8] Schuett DJ Bomar JD Pennock AT. Pelvic apophyseal avulsion fractures: a retrospective review of 228 cases. J Pediatr Orthop. (2015) 35:617–23. 10.1097/BPO.0000000000000328 25321882

[9] Kautzner J Trc T Havlas V. Comparison of conservative against surgical treatment of anterior-superior iliac spine avulsion fractures in children and adolescents. Int Orthop. (2014) 38:1495–8. 10.1007/s00264-014-2323-0 24695975 PMC4071503

[10] Schoensee SK Nilsson KJ. A novel approach to treatment for chronic avulsion fracture of the ischial tuberosity in three adolescent athletes: a case series. Int J Sports Phys Ther. (2014) 9:974–90. Available from: http://www.ncbi.nlm.nih.gov/pubmed/25540712%0Ahttp://www.pubmedcentral.nih.gov/articlerender.fcgi?artid=PMC4275201 25540712 PMC4275201

[11] Khemka A Raz G Bosley B Ludger G Al Muderis M. Arthroscopically assisted fixation of the lesser trochanter fracture: a case series. J Hip Preserv Surg. (2014) 1:27–32. 10.1093/jhps/hnu006 27011799 PMC4765264

[12] Eberbach H Hohloch L Feucht MJ Konstantinidis L Südkamp NP Zwingmann J. Operative versus conservative treatment of apophyseal avulsion fractures of the pelvis in the adolescents: a systematical review with meta-analysis of clinical outcome and return to sports. BMC Musculoskelet Disord. (2017) 18:162. 10.1186/s12891-017-1527-z 28420360 PMC5395880

[13] Moher D Liberati A Tetzlaff J Altman DG. Preferred reporting items for systematic reviews and meta-analyses: the PRISMA statement. PLoS Med. (2009) 6:e1000097. 10.1371/journal.pmed.1000097 19621072 PMC2707599

[14] Serbest S Tosun HB Tiftikçi U Oktas B Kesgin E. Anterior inferior iliac spine avulsion fracture. Medicine. (2015) 94:e562. 10.1097/MD.0000000000000562 25700329 PMC4554161

[15] Uzun M Alpan B Özger H. Avulsion fractures involving the straight and reflected heads of the rectus femoris. HIP Int. (2014) 24:206–9. 10.5301/hipint.5000110 24500831

[16] Cai W Xie Y Su Y. Comparison of non-surgical and surgical treatment using absorbable screws in anterior-superior iliac spine avulsion fractures with over 1.5 cm displacement. Orthop Traumatol Surg Res. (2020) 106:1299–304. Available from: 10.1016/j.otsr.2020.02.014 32409270

[17] Kosanović M Brilej D Komadina R Buhanec B Pilih IA Vlaović M. Operative treatment of avulsion fractures of the anterior superior iliac spine according to the tension band principle. Arch Orthop Trauma Surg. (2002) 122:421–3. 10.1007/s00402-002-0396-5 12442175

[18] Willinger L Schanda JE Lorenz S Imhoff AB Buchmann S. Surgical treatment of two adolescent athletes with dislocated avulsion fracture of the anterior superior iliac spine (ASIS). Arch Orthop Trauma Surg. (2017) 137:173–7. 10.1007/s00402-016-2596-4 27866232

[19] Pogliacomi F Calderazzi F Paterlini M Ceccarelli F. Surgical treatment of anterior iliac spines fractures: our experience. Acta Biomed. (2014) 85:52–8. 25409719

[20] Ruffing T Rückauer T Bludau F Hofmann A Muhm M Suda AJ. Avulsion fracture of the lesser trochanter in adolescents. Injury. (2018) 49:1278–81. 10.1016/j.injury.2018.04.030 29747942

[21] Volpi A Matzko C Feghhi D Matheney T Bharam S. Conservative treatment of avulsion injuries of the lesser trochanter in adolescent athletes. Cureus. (2021) 13:e15638. 10.7759/cureus.15638 34306849 PMC8278968

[22] Otto A Banke IJ Mehl J Beitzel K Imhoff AB Scheiderer B. Retrograde fixation of the lesser trochanter in the adolescent: new surgical technique and clinical results of two cases. Arch Orthop Trauma Surg. (2019) 139:537–45. 10.1007/s00402-018-3091-x 30535582

[23] Ferlic PW Sadoghi P Singer G Kraus T Eberl R. Treatment for ischial tuberosity avulsion fractures in adolescent athletes. Knee Surg Sport Traumatol Arthrosc. (2014) 22:893–7. 10.1007/s00167-013-2570-4 23793970

[24] Biedert RM. Surgical management of traumatic avulsion of the ischial tuberosity in young athletes. Clin J Sport Med. (2015) 25:67–72. 10.1097/JSM.0000000000000088 24662573

[25] Best R Meister A Huth J Becker U Meier M. Surgical repair techniques, functional outcome, and return to sports after apophyseal avulsion fractures of the ischial tuberosity in adolescents. Int Orthop. (2021) 45:1853–61. 10.1007/s00264-021-04959-w 33963885 PMC8266717

[26] Li X Xu S Lin X Wang Q Pan J. Results of operative treatment of avulsion fractures of the iliac crest apophysis in adolescents. Injury. (2014) 45:721–4. 10.1016/j.injury.2013.10.005 24246879

[27] Sinikumpu JJ Hetsroni I Schilders E Lempainen L Serlo W Orava S. Operative treatment of pelvic apophyseal avulsions in adolescent and young adult athletes: a follow-up study. Eur J Orthop Surg Traumatol. (2018) 28:423–9. 10.1007/s00590-017-2074-x 29159479

[28] McKinney BI Nelson C Carrion W. Apophyseal avulsion fractures of the hip and pelvis. Orthopedics. (2009) 32:42. 10.3928/01477447-20090101-12 19226032

